# Post-functionalization of drug-loaded nanoparticles prepared by polymerization-induced self-assembly (PISA) with mitochondria targeting ligands

**DOI:** 10.3762/bjoc.17.148

**Published:** 2021-09-03

**Authors:** Janina-Miriam Noy, Fan Chen, Martina Stenzel

**Affiliations:** 1 School of Chemistry, University of New South Wales, Sydney NSW 2052, Australia

**Keywords:** micelle, mitochondria, phosphorylcholine, PISA, polymerization-induced self-assembly

## Abstract

Herein, the postfunctionalization of different non-fouling PISA particles, prepared from either poly(oligo ethylene glycol methyl ether methacrylate) (pPEGMA) and the anticancer drug PENAO (4-(*N*-(*S*-penicillaminylacetyl)amino)phenylarsenonous acid) or zwitterionic 2-methacryloyloxyethyl phosphorylcholine (MPC) and PENAO were reported. Both PISA particles were reacted with triphenylphosphonium (TPP) as mitochondria targeting units in order to evaluate the changes in cellular uptake or the toxicity of the conjugated arsenic drug. Attachment of TPP onto the PISA particles however was found not to enhance the mitochondrial accumulation, but it did influence overall the biological activity of pMPC-based particles in 2D and 3D cultured sarcoma SW982 cells. When TPP was conjugated to the pMPC PISA particles more cellular uptake as well as better spheroid penetration were observed, while TPP on PEG-based PISA had only little effect. It was hypothesized that TPP on the micelle surface may not be accessible enough to allow mitochondria targeting, but more structural investigations are required to elucidate this.

## Introduction

Targeting mitochondria is a promising strategy for the development of new anticancer drugs [1]. Among them, organoarsenical drugs have shown great promise as these drugs inhibit the function of mitochondria while showing overall reduced systemic toxicity. PENAO (4-(*N*-(*S*-penicillaminylacetyl)amino)phenylarsenonous acid) [2–3], a trivalent arsenical drug, was developed by Hogg and co-workers and is currently in clinical trials [4]. PENAO triggers cell apoptosis by targeting the ANT protein, which is located in the mitochondria. More specifically, PENAO binds to the thiols located on the ANT peptide loops Cys^57^ and Cys^257^, which results in the formation of stable cyclic dithioarsinite complexes [5]. Arsenic drugs do not only bind to mitochondrial protein, but also chelates other cysteine-containing species. Several hundred good binding sites for trivalent arsenicals in each organ have been proposed [6–7], and more than 50 arsenic-binding proteins could be identified and analysed by Zhang et al. [8] and Yan et al. [9] using *p*-phenylarsenoxide-based agents. This can also lead to deactivation of the drug as these organoarsenic drugs react readily with blood proteins, in particular transferrin [10]. In order to limit premature inactivation of the arsenic drugs, a range of nanoparticles have been developed to enhance stability, thus increase activity [11–12]. These nanoparticles have been decorated with targeting ligands to enhance the accumulation of the drug in cancer tissue. The activity of arsenic drugs could, however, also be enhanced by targeting mitochondria inside the cells, the target of action of arsenic drugs.

To the current date, one of the most effective methods to specifically target the mitochondria is the covalent attachment of mitochondrial penetrating molecules to carrier systems [13–14]. Several mitochondriotropic moieties such as dequalinium (DQA), triphenylphosphonium (TPP), and mitochondrial penetrating peptides and proteins have been linked to nanotechnology. DQA and TPP are both cationic and lipophilic molecules and therefore able to easily pass the mitochondrial membrane [15–19]. TPP is the most-studied mitochondrial targeting agent and has shown to accumulate 1000 times more in the mitochondrial matrix than in the cytosol [20]. TPP derivatives were attached to PEGylated poly(ε-caprolactone) (PCL), and corresponding micellar formulation enabled the delivery of coenzyme Q10 (CoQ10) to the mitochondria [21]. In addition, PEGylated TPP-conjugated poly(lactic-*co*-glycolic acid) nanoparticles have shown to increase mitochondrial accumulation when more positive zeta potentials were obtained [22], and when the TPP-nanoparticles were loaded with drugs, significantly better antitumour activities were acquired [23–27], highlighting TPP’s desirable influence on the therapeutic efficiency.

Micelles are ideal carriers when designing a nanoparticle with an abundance of functional groups on the surface, which could be conjugated with targeting ligands such as TPP. Moreover, they have already been used to successfully deliver arsenic drugs [28–32], highlighting their suitability as a potential vehicle to deliver these drugs. Disadvantage is however the multistep procedure to generate micelles ranging from the synthesis of block copolymers to the self-assembly into micelles, often in low dilution, making the process inefficient. Micelles obtained by the PISA process can in contrast generate large amounts of nanoparticles in a reproducible manner. PISA nanoparticles have been frequently investigated as drug carriers. The challenge is often how to entrap the drug during the self-assembly process. Addition of drugs during the PISA process is possible [33–34], but it needs to be taken into account that the drug can interfere with the block copolymer aggregation. Alternatively, the use of reactive polymers for the polymerization creates a functional anchor to enable post-functionalization of the PISA nanoparticles with drugs such as doxorubicin [35–36]. In a different strategy, drug conjugated monomers can be directly used in the polymerization, eliminating the post-modification step [28,37–39].

In order to use micelles, prepared by PISA or traditional techniques, for targeting specific receptors, it is necessary that these materials are low-fouling, thus repel non-specific protein absorption. Su et al. [40] investigated the effects of a protein corona on active and passive targeting using 20 different types of PEGylated gold nanoparticles, which were decorated with cyclic RGD (arginylglycylaspartic acid) peptides. As a result, the active targeting efficiency on a protein covered nanoparticle was significantly reduced compared to a non-protein bounded nanoparticle. Stealth like nanoparticle surfaces, such as surfaces covered with polyethylene glycol or phosphorylcholine, are therefore attractive for biological applications, as they have the ability to repel proteins and therefore reduce the possibility of macrophage clearance [41]. Therefore, in this study we will use poly(2-methacryloyloxyethyl phosphorylcholine) (pMPC) and poly(oligo ethylene glycol methyl ether methacrylate) (pPEGMA) as protein-repellent polymers in order to ensure that the formation of the protein corona is reduced.

In earlier studies we have shown the polymerization-induced self-assembly is an excellent tool to generate nanoparticles with conjugated PENAO in situ [28]. We described how the zwitterionic 2-methacryloyloxyethyl phosphorylcholine (MPC), copolymerized with 4-(*N*-(*S*-penicillaminylacetyl)amino)phenylarsonous acid methacrylamide, which is PENAO reacted with a polymerizable group, can be used as a stabilizing block for the subsequent PISA reaction with methyl methacrylate (MMA) [42]. We also showed how small changes to the stabilizing block can cause changes in the self-assembly process [43]. Moreover, a second nanoparticle based on poly(oligo ethylene glycol methyl ether methacrylate) (pPEGMA) was generated as both polymers, pPEGMA and pMPC display protein-repellent properties [44–45]. In this paper, we aimed at comparing the effect of nanoparticles with and without triphenylphosphonium (TPP) as mitochondria targeting units. In order to keep the aggregation number, and therefore the size of the nanoparticle, constant, we opted to post-functionalize the nanoparticles with TPP to facilitate the study of the effect of TPP only. Aim of this project is to explore if the attachment of mitochondria targeting ligands can enhance the activity of PENAO using the nanoparticles that were recently described in our group [28,42].

## Results and Discussion

### Synthesis of Micelles by PISA

Initially two nanoparticles were prepared that had comparable particle sizes, which enabled not only comparison of nanoparticles with and without TPP, but also the effect of the shell material, pPEGMA and pMPC. The synthesis of p(MPC-*co*-PENAO)-*b*-p(MMA) and p(PEGMA-*co*-PENAO)-*b*-p(MMA) nanoparticles has been described in our earlier publication [28]. Initially, the monomer 4-(*N*-(*S*-penicillaminylacetyl)amino)phenylarsonous acid methacrylamide was prepared by reaction of PENAO with methacylic anhydride [29] and then copolymerized with MPC or PEGMA using CPADB-OH (2-hydroxyethyl 4-cyano-4-((phenylcarbonothioyl)thio)pentanoate) as RAFT agent. This RAFT agent is based on the well-studied 4-cyano-4-((phenylcarbonothioyl)thio)pentanoic acid, but it was modified with ethylene glycol. During the polymerization of the water-soluble polymers, fluorescein *O*-methacrylate was added at a ratio of CTA to fluorescent monomer of 1:0.3. This means more that three out of ten polymer chains will be labelled, which is sufficient for cell work. The water-soluble polymer was then chain-extended via PISA using MMA at a various feed ratio of MMA and RAFT agent using earlier procedures [42], resulting in nanoparticles with hydroxy functionalities located on the surface. In earlier studies it was observed that shorter MPC blocks result in more bioactive PISA nanoparticles [28]. Therefore, the relative short MPC block (p(MPC_17_-*co*-PENAO_4_), **MP2** ) was selected, which was reacted with MMA in water–methanol to yield nanoparticles of around 76 nm (Table 1). In order to identify a matching nanoparticle of similar size with a PEG surface, a range of polymerizations with various concentrations had to be carried out. As described earlier, a range of PISA particles had to be prepared in order to generate two particles of similar size [42]. In general, it was necessary to use longer blocks based on PEGMA in order to achieve similar particles sizes, which was discussed in detail elsewhere [42]. Here, the best two candidates as discussed in reference [42] were used.

**Table 1:** Summary of the pPEGMA and pMPC block copolymers prepared by PISA at 70 °C including the hydrodynamic diameter *D*_h_ and particle size distribution PdI obtained by DLS and the drug loading content (DLC) calculated using DLC = m(PENAO)/[m(PENAO) + m(polymer)]. The table here contains the two nanoparticles used in this work. The full table describing several nanoparticles can be found in [42].

Particles	[MMA]:[PP/MP]:[I]	Time (h)	Conv.^a^ (%)	*D*_h_ (nm)	PdI	DLC (%)

p(PEGMA_63_-*co*-PENAO_7_)-*b*-p(MMA)_2838 _**PPM-NP4**	5000:1:0.2 **PP3**	4.5	57	85.4 ± 0.9	0.067	0.79
p(MPC_17_-*co*-PENAO_4_)-*b*-p(MMA)_1485 _**MPM-NP2**	1500:1:0.2 **MP2**	6	99	75.9 ± 1.2	0.094	0.89

^a^Determined by ^1^H NMR spectroscopy.

Both systems, p(PEGMA_63_-*co*-PENAO_7_)-*b*-p(MMA)_2838_ **PPM-NP4** and p(MPC_17_-*co*-PENAO_4_)-*b*-p(MMA)_1485_ **MPM-NP2**, resulted in spherical core-shell nanoparticles with sizes of around 80 nm (Table 1). It is evident that it is not possible to compare two nanoparticles with similar repeating units or similar molecular weight as both polymers, pMPC and p(PEGMA), influence the PISA polymerization in different ways. However, the chosen particles are comparable in size and drug loading content (DLC, Table 1). These nanoparticles were already described in [42], but they are included in this publication for convenience of the reader.

### Modification with TPP

**Figure 1:**
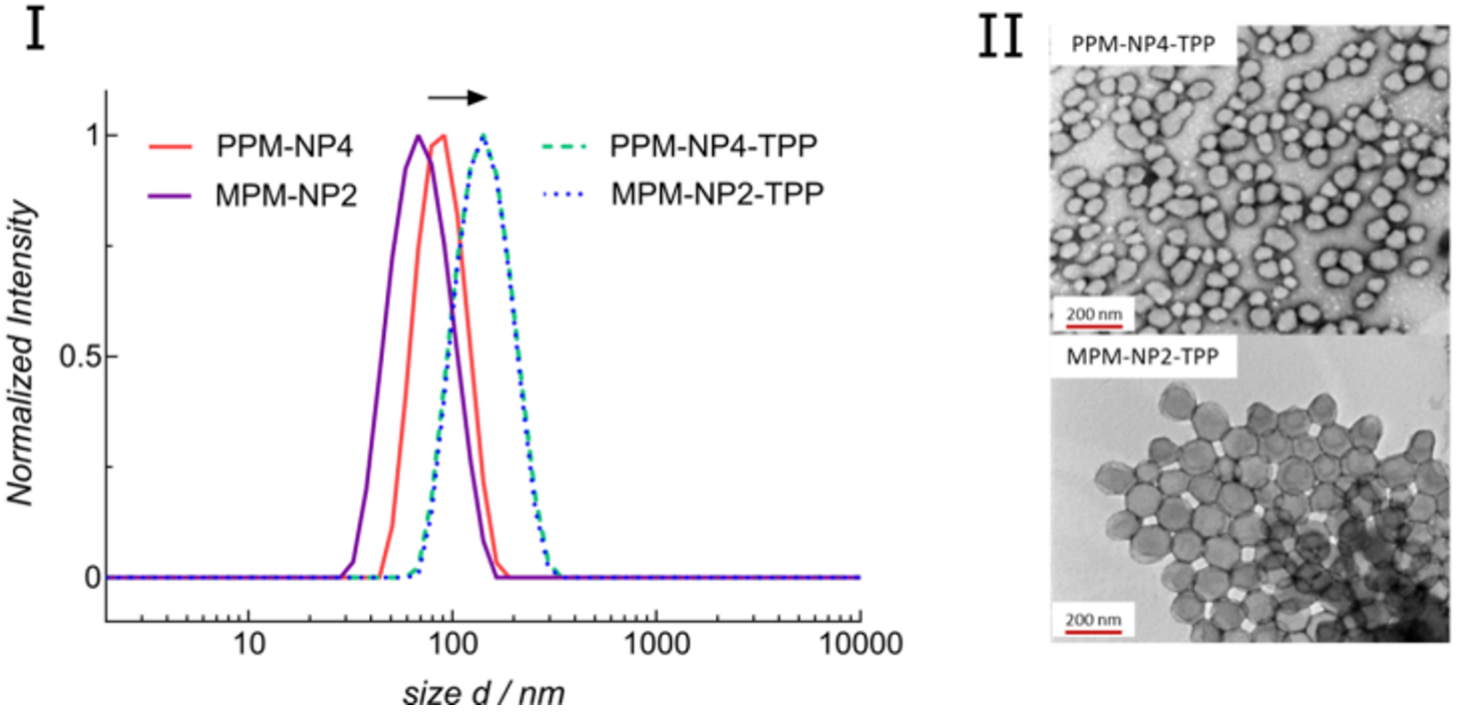
(I) DLS of **PPM-NP4**, **MPM-NP2**, **PPM-NP4-TPP** and **MPM-NP2-TPP** and (II) TEM of **PPM-NP4-TPP** and **MPM-NP2-TPP** (stained with uranyl acetate). Note: The curves of **PPM-NP4-TPP** and **MPM-NP2-TPP** are overlapping.

The PISA particles with excess hydroxy groups on the surface were subsequently modified with the water-soluble TPP derivate, TPP-COOH ((4-carboxybutyl)triphenylphosphonium bromide), which was chosen as a target agent for the herein conducted studies, and covalently coupled to **PPM-NP** and **MPM-NP** particles. The reaction was conducted in aqueous medium, using 5 equiv excess of TPP-COOH to RAFT-end group. The pH was first adjusted to 5.2 at the start of the reaction and after 45 min tuned to 8.3 to allow maximum conjugation efficiency. After removing excess TPP-COOH and coupling reagents via dialysis in Milli-Q water, the particles were adjusted to 4 mg mL^−1^ and analysed using DLS experiments and TEM microscopy (Figure 1). The attachment of TPP-COOH to the micelle variants resulted in an increase in hydrodynamic diameter from approximately 85 nm to 136 nm for the PEG micelles and from 75 nm to 138 nm for the zwitterionic counterparts. However, no major change in dispersities was detected and well-defined nanoparticles (PdI < 0.078) were formed. Furthermore, the surface charge became less negative for both particle systems (**PPM-NP4** = −14.1 mV, **PPM-NP4-TPP** = −4.7 mV; **MPM-NP2** = −15.6 mV, **MPM-NP2-TPP** = −5.1 mV) (Table 2), confirming the successful attachment of the positively charged mitochondria agent.

**Table 2:** Summary of **PPM-NP4** and **MPM-NP2** before and after attachment of TPP-COOH.

Particles	*D*_h_^a^ (nm)	PdI^a^	*D*_h_^b^ (nm)	PdI^b^	ζ^a^ (mV)	ζ^b^ (mV)	cmc^a^ (µM)	cmc^b^ (µM^1^)

**PPM-NP4**/**PPM-NP4-TPP**	85.4 ± 0.9	0.067	136.3 ± 0.5	0.078	−14.1	−4.7	2.3	6.5
**MPM-NP2**/**PPM-NP2-TPP**	75.9 ± 1.2	0.094	137.5 ± 1.5	0.074	−15.6	−5.1	5.6	14.6

^a^Before TPP attachment. ^b^After TPP attachment.

**Figure 2:**
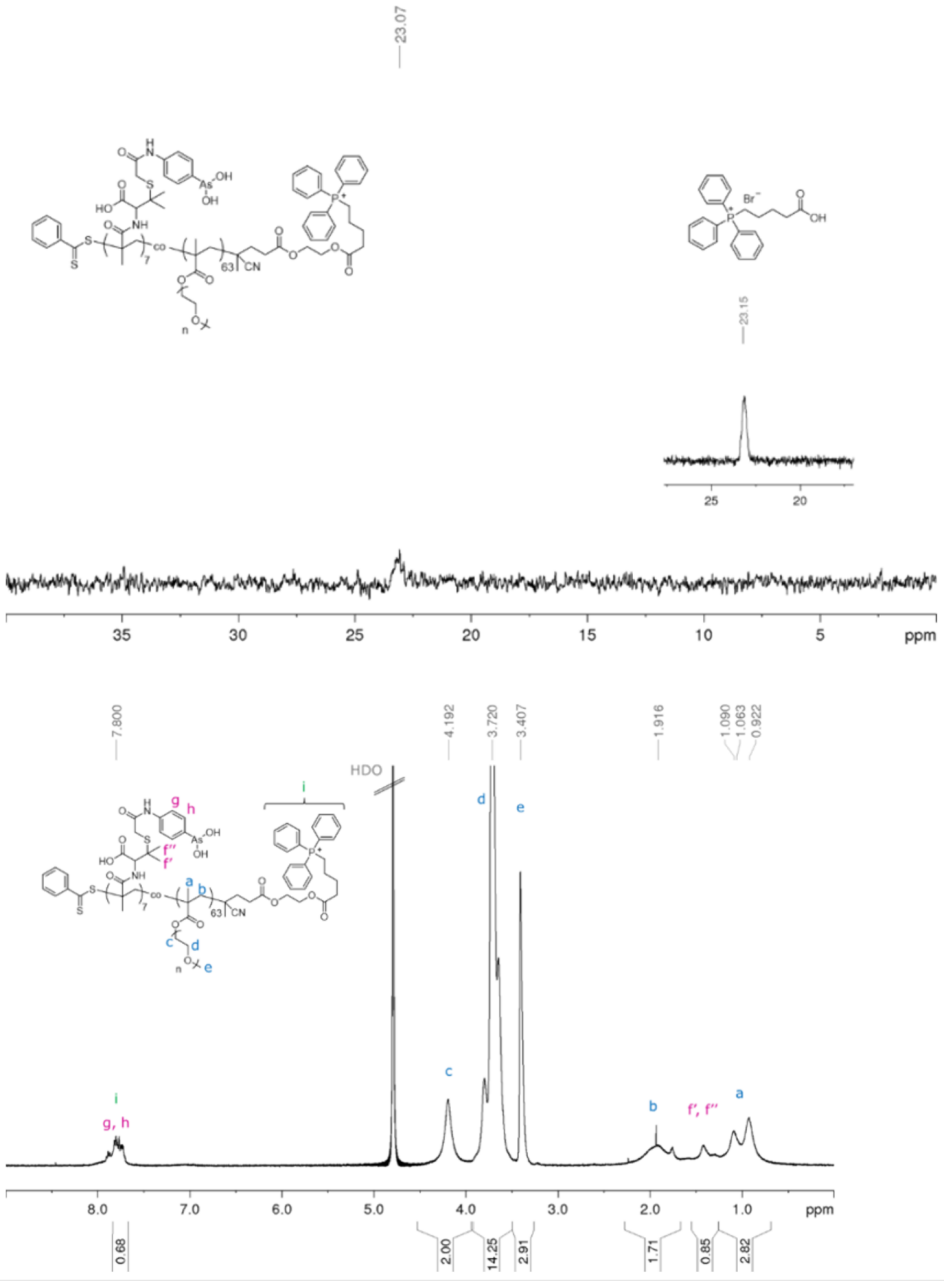
Representative ^31^P NMR (top) and ^1^H NMR (bottom) spectrum of **PP3-TPP** conjugation product in D_2_O.

No quantitative elucidation via NMR spectroscopy or UV–vis measurement was possible due to the extremely high molecular weights of the particles. The NMR spectra in D_2_O showed only very broad peaks of low intensity and no aromatic peaks, indicative of TPP attachment, were detected. This could of course mean that TPP has either not reacted, is buried inside the shell or NMR is not sufficiently sensitive to detect the attachment of an end group in large nanoparticles. In any case, this approach was inconclusive. Hence, the copolymer **PP3** (p(PEGMA_63_-*co*-PENAO_7_) was used as a model compound and reacted with TPP-COOH under the same conditions as described above. The conjugation product was analysed using ^1^H and ^31^P NMR spectroscopy. The ^31^P NMR spectrum (Figure 2a) shows a peak at 23.07 ppm which belongs to the phosphorus of the triphenylphosphonium moiety. Furthermore, the additional peaks in the region between 7.5–8.2 ppm in the ^1^H NMR spectrum (Figure 2b) can be associated to the phenyl protons of the TPP residue. The integration cannot be calculated using the methylene signal adjacent to the ester of PEGMA at δ = 4.1 ppm as this peak will overlap with the ethylene glycol spacer to TPP. Instead, the methyl signal 3.4 ppm was used. As the polymer has 63 PEGMEMA repeating units, the intensity of this peak is 189, which should be equivalent to the 15 aromatic peaks of TPP. This signal overlaps with 7 × 4 aromatic peaks belonging to PENAO. Care needs to be taken here as this is also the region of the aromatic group of the RAFT agent although there is no direct overlap. Full TPP endchain modification therefor equates to an intensity ratio of 43 (δ = 7.5–8.2 ppm) to 189 (δ = 3.4 ppm), which is equivalent to the signal intensity of 2.91 (δ = 3.4 ppm) to 0.68 (δ = 7.5–8.2 ppm) shown in Figure 2, suggesting complete modification.

**Scheme 1:**
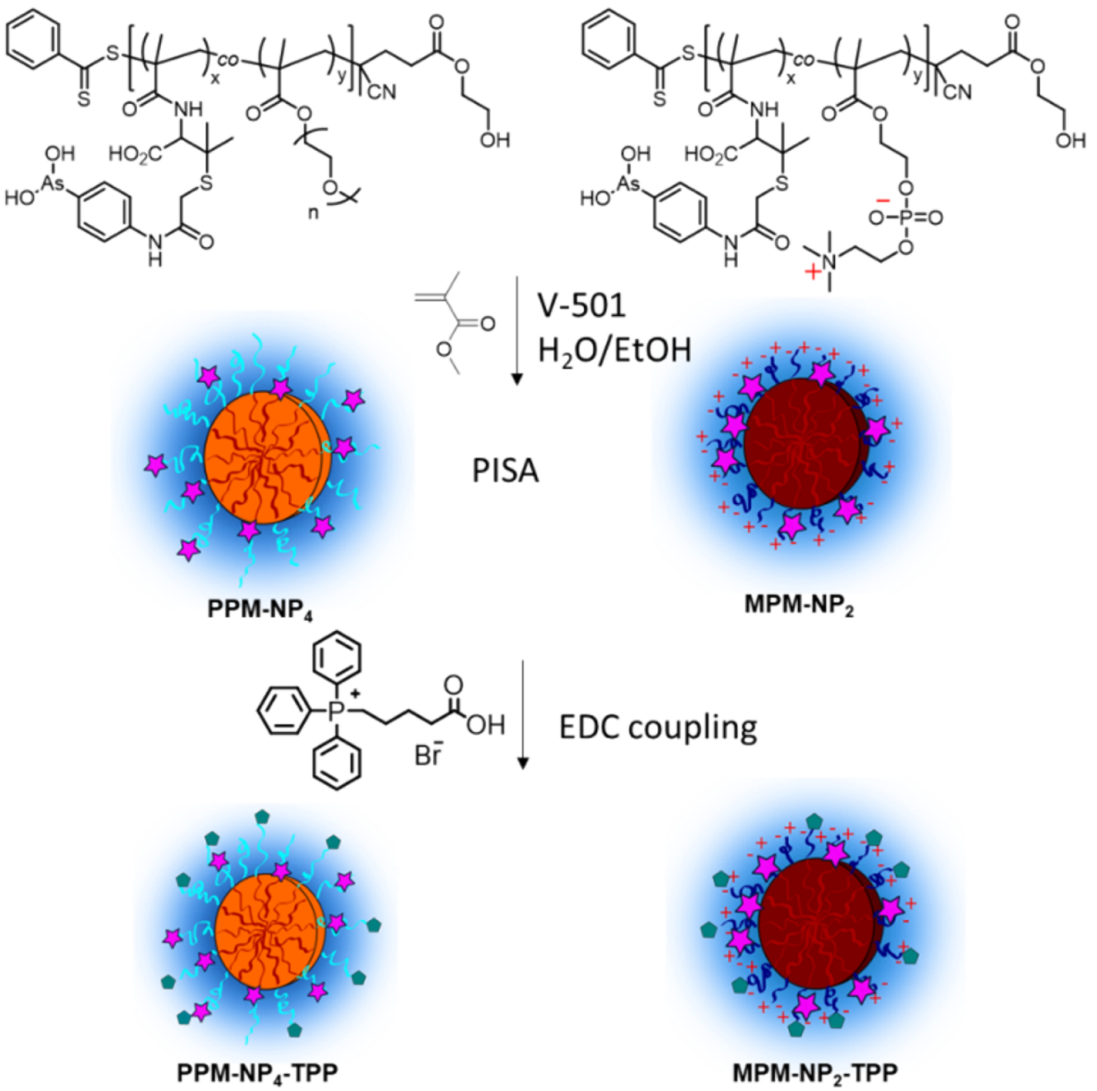
Synthesis of TPP-based PISA particles based on zwitterionic 2-methacryloyloxyethyl phosphorylcholine (**PPM-NP4-TPP**) and poly(oligo ethylene glycol methyl ether methacrylate) (pPEGMA) with a pMMA core and pendant PENAO drugs (pink stars). The synthesis of the nanoparticles was described earlier [28,42], here, the postmodification with TPP was explored. Parts of the scheme were adapted from ref. [28], © 2018 American Chemical Society. This content is not subject to CC BY 4.0.

The integration for the peak at the region (g, h and i) increased by 0.26 in number, suggesting that approximately one TPP group per chain was attached. The model reaction using **PP3** revealed that the conjugation of TPP-COOH to the polymer proceeded in a quantitative manner and can be used as a good indication for the reaction efficiency between the hydroxy residue of the RAFT-end group and the carboxylic group of the TPP molecule. It was assumed that the particle systems show similar reactivity profiles and resulted in high TPP attachment as depicted in Scheme 1. However, that still slightly negative zeta potentials were obtained, indicates that somewhat lower conjugation products were achieved. This is not surprising as the introduction of positive charges on the surface will introduce stress to the system due to strong repulsive forces, thus the complete reaction is prevented. At this stage, it is difficult to determine how many end groups were actually modified as typical techniques such as NMR are not sensitive enough to detect small changes on the polymer end groups. At this point it can be argued that it would be easier to prepare a TPP-modified RAFT agent as this would be the only way to ensure high end group fidelity. This is of course true, but it also needs to be considered that the presence of the end group will influence the PISA process and results in aggregation numbers and particle sizes that are very different to the ones obtained with unmodified RAFT agent.

Due to the observed change in size after ligand attachment, the stability of the particles to disassembly was analysed by measuring the scattering intensity for different polymer concentrations (*c* = 0–100 µM) and the critical micelle concentration (cmc) values were determined from the intercept point of the linear regressions as shown in Supporting Information File 1, Figure S1. **PPM-NP4** demonstrated a slightly lower cmc value (cmc = 2.3 µM) compared to **MPM-NP2** (cmc = 5.6 µM), thus is the most stable. After TPP conjugation to the surface of the nanoparticles, the stability for both micelle variants decreased by approximately 2.5-fold (Table 2). Furthermore, the more neutral zeta potentials of the TPP-conjugated particles influences the stability of particles to aggregation. High zeta potentials – positive or negative – result typically in less aggregated particle systems [20,46].

### Biological characterization

**Figure 3:**
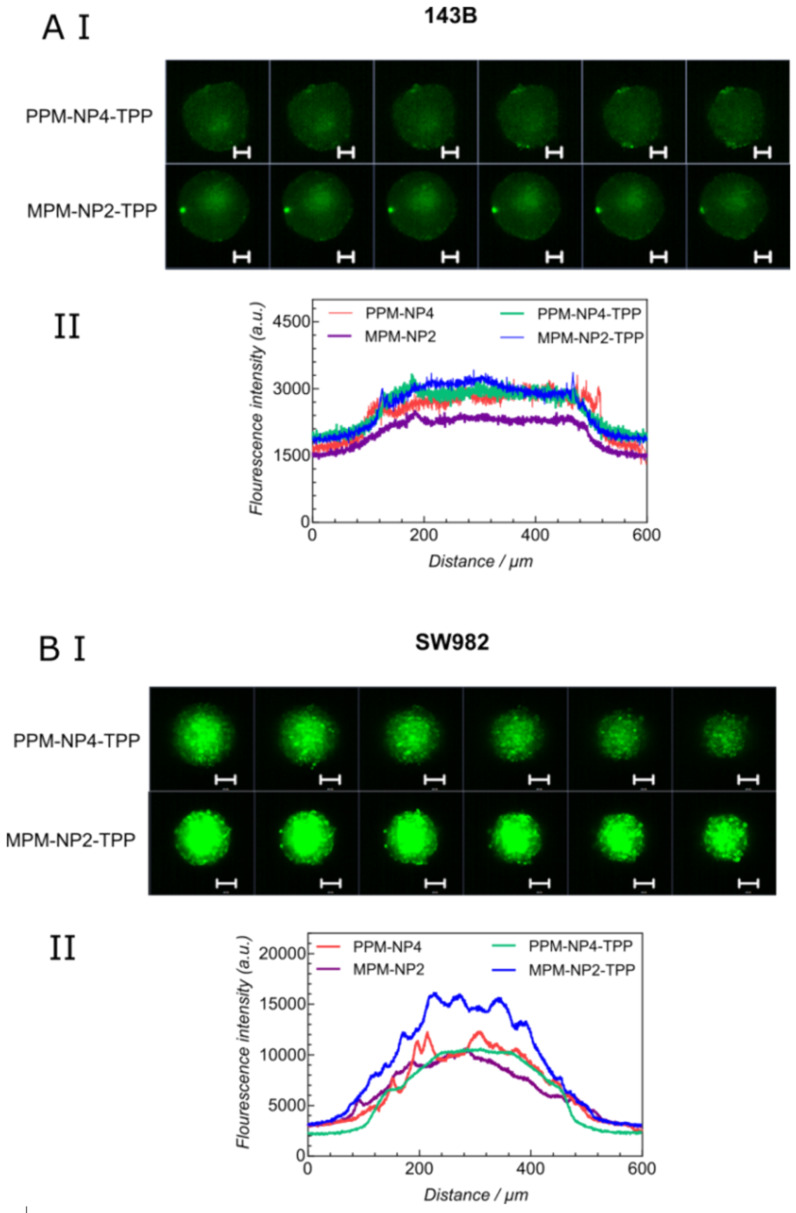
Penetration of **PPM-NP4-TPP** and **MPM-NP2-TPP** micelles and fluorescence intensity profile on (A I, II) 143B and (B I, II) SW982 spheroids (scale bars = 300 µm). The results of the nanoparticles without TPP have been reported earlier [42]. This figure was adapted with permission from [42], Noy et al., Direct Comparison of Poly(ethylene glycol) and Phosphorylcholine Drug-Loaded Nanoparticles In Vitro and In Vivo, *Biomacromolecules* **2020,** *21,* 2320–2333. Copyright 2020 American Chemical Society. This content is not subject to CC BY 4.0.

The mitochondria target containing nanoparticles **PPM-NP4-TPP** and **MPM-NP2-TPP** were further analysed using 3D spheroid tumour models [47]. The samples were incubated with 143B and SW982 cells and the penetration profiles of the micelles were investigated using laser scanning confocal microscopy. After 3 hours of incubation, the penetration profiles of the micelles into the spheroids can be visualized as seen in Figure 3. The PEG-based micelle, **PPM-NP4-TPP**, reveals no significant change in fluorescence compared to the non TPP-conjugated micelle, **PPM-NP4** (green and red curves respectively, Figure 3A II and B II)). No dominant improvement in spheroid penetration could therefore be detected. Interestingly, for **MPM-NP2-TPP** significant higher fluorescence intensities were measured, when the TPP-micelle was subjected to 143B and SW982 MCTS (Figure 3). As a result, high spheroid uptake and deep spheroid penetration were observed, particularly into the soft tissue sarcoma spheroids, indicating that the TPP conjugation improved the internalization into tumours for **MPM-NP2-TPP**, despite being slightly less stable than the non-conjugate micelles.

**Figure 4:**
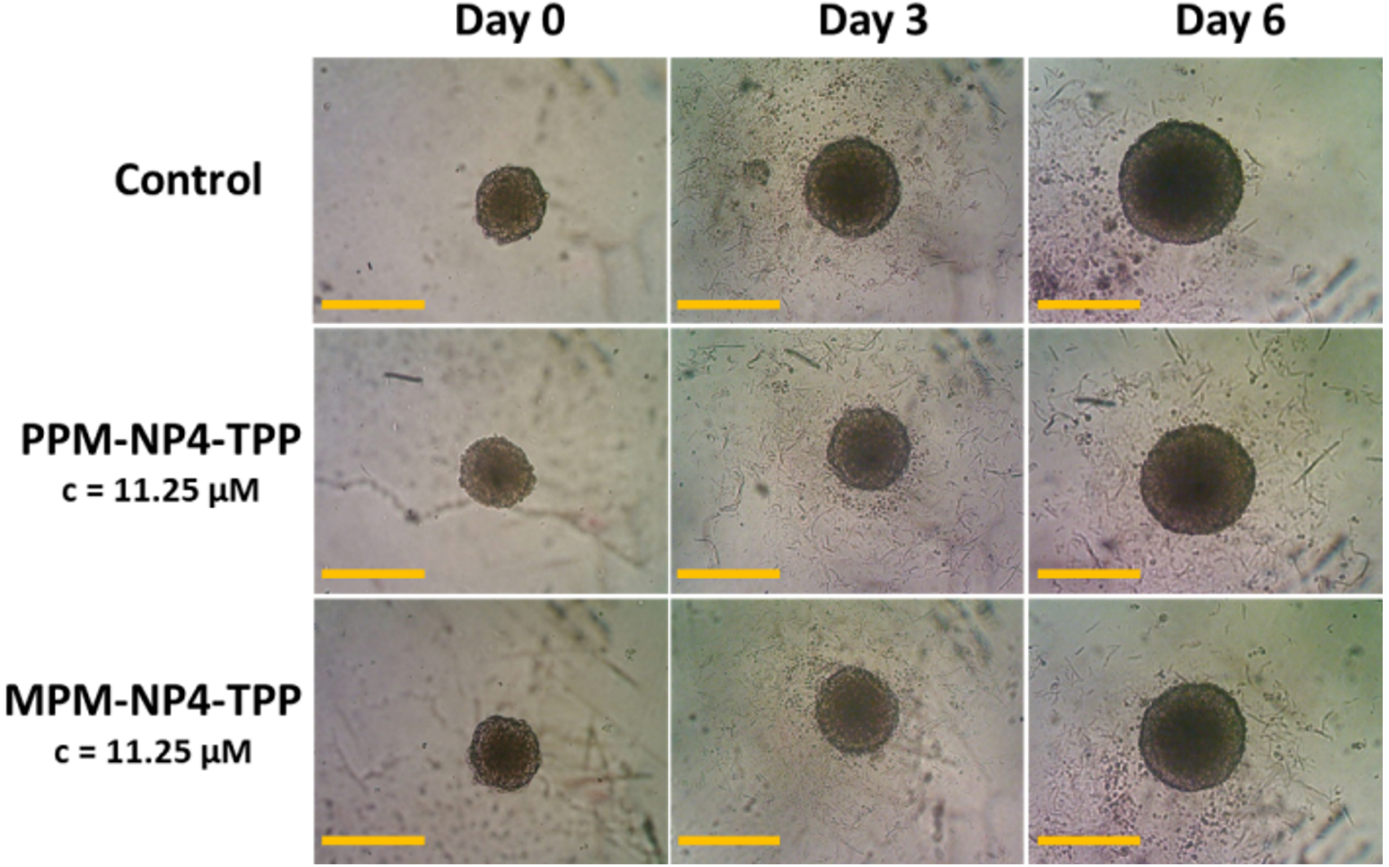
Growth effects of **PPM-NP4-TPP** and **MPM-NP2-TPP** on SW982 spheroids after 3 and 6 days of incubation (*c*(drug) = 11.25 µM, scale bar 300 µm).

**Figure 5:**
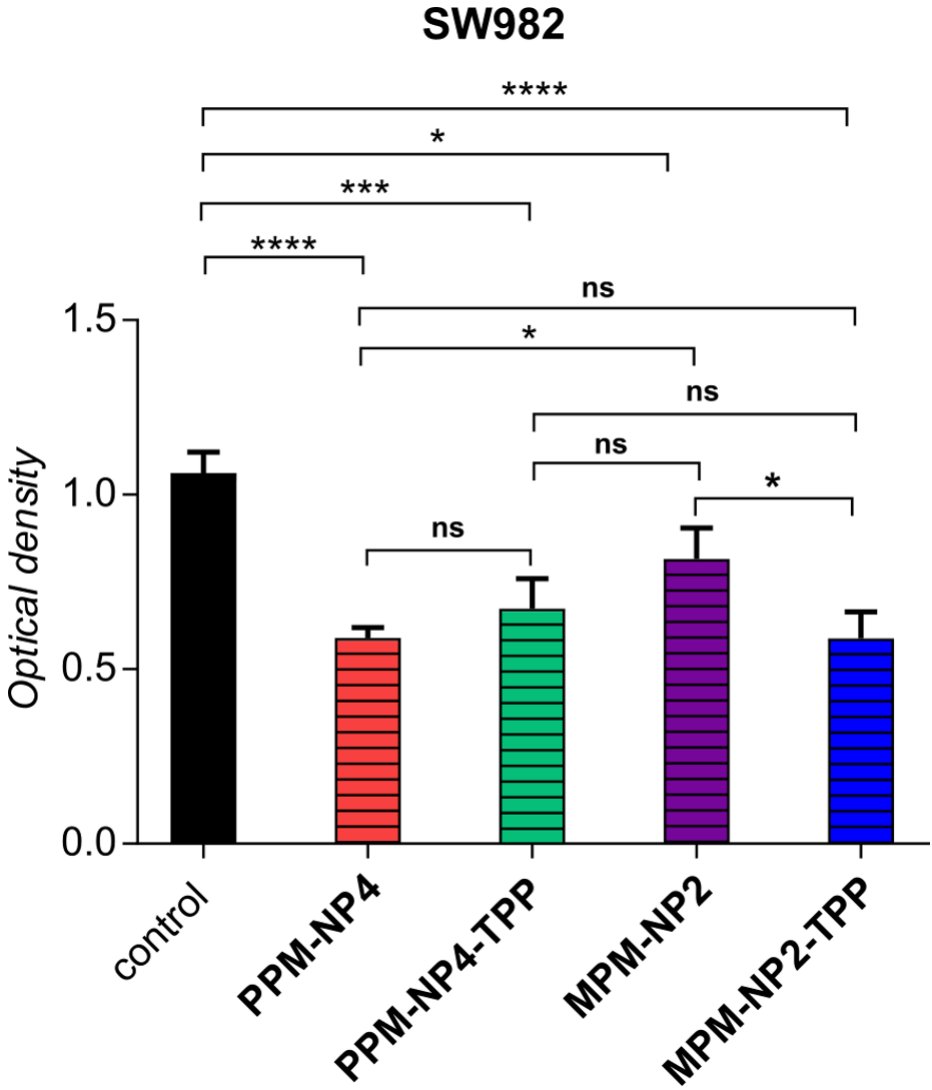
Cell viability of SW982 spheroids after 6 days treatment with **PPM-NP4**, **PPM-NP-TPP**, **MPM-NP2** and **MPM-NP2-TPP** (*c*(drug) = 11.25 µM). Data represent means ± SD, *n* = 6. *, significant difference, *P* < 0.05; **, significant difference, *P* < 0.01; ***, significant difference, *P* < 0.001; ****, significant difference, *P* < 0.0001.

The TPP-particles were further investigated for spheroid growth inhibition using the SW982 MCTS model. The spheroids were treated with the nano-objects at a drug concentration *c* = 11.25 µM and the size and morphology of the MCTS were traced after 3 and 6 days. The chosen concentration was based on IC_50_ values of various PENAO formulations measured in earlier works, which typically ranged around this value. Both treated spheroids led to smaller tumour MCTS compared to the control (Figure 4). No dominant optical difference could be detected between the nanoparticle samples. Therefore, the optical cell density of **PPM-NP4-TPP** and **MPM-NP2-TPP** treated spheroids was elucidated via the APH assay after 6 days of treatment and compared with each other and with the previous obtained optical densities of **PPM-NP4** and **MPM-NP2** treated MCTS (Figure 5). As already seen in the images in Figure 4, the cell viability of the micelle-treated spheroids is lower than that of the control. No improvement in treatment was achieved for the **PPM-NP4-TPP** micelles, which is in agreement with the 3D penetration studies. On the other hand, the zwitterionic **MPM-NP2-TPP** particles induced more cell death and represent a lower optical cell density for SW982 MCTS. This outcome reveals that the attachment of TPP to zwitterionic particles accelerated its anticancer performance in terms of spheroid uptake and tumour growth inhibition, while no anticancer enhancement was detected when the PEGylated micelle was conjugated with a mitochondrial agent. In conclusion, the conjugation of TPP to **MPM-NP2** gave the zwitterionic drug particles similar anticancer efficiency than that of the non-conjugated **PPM-NP4** micelles.

**Figure 6:**
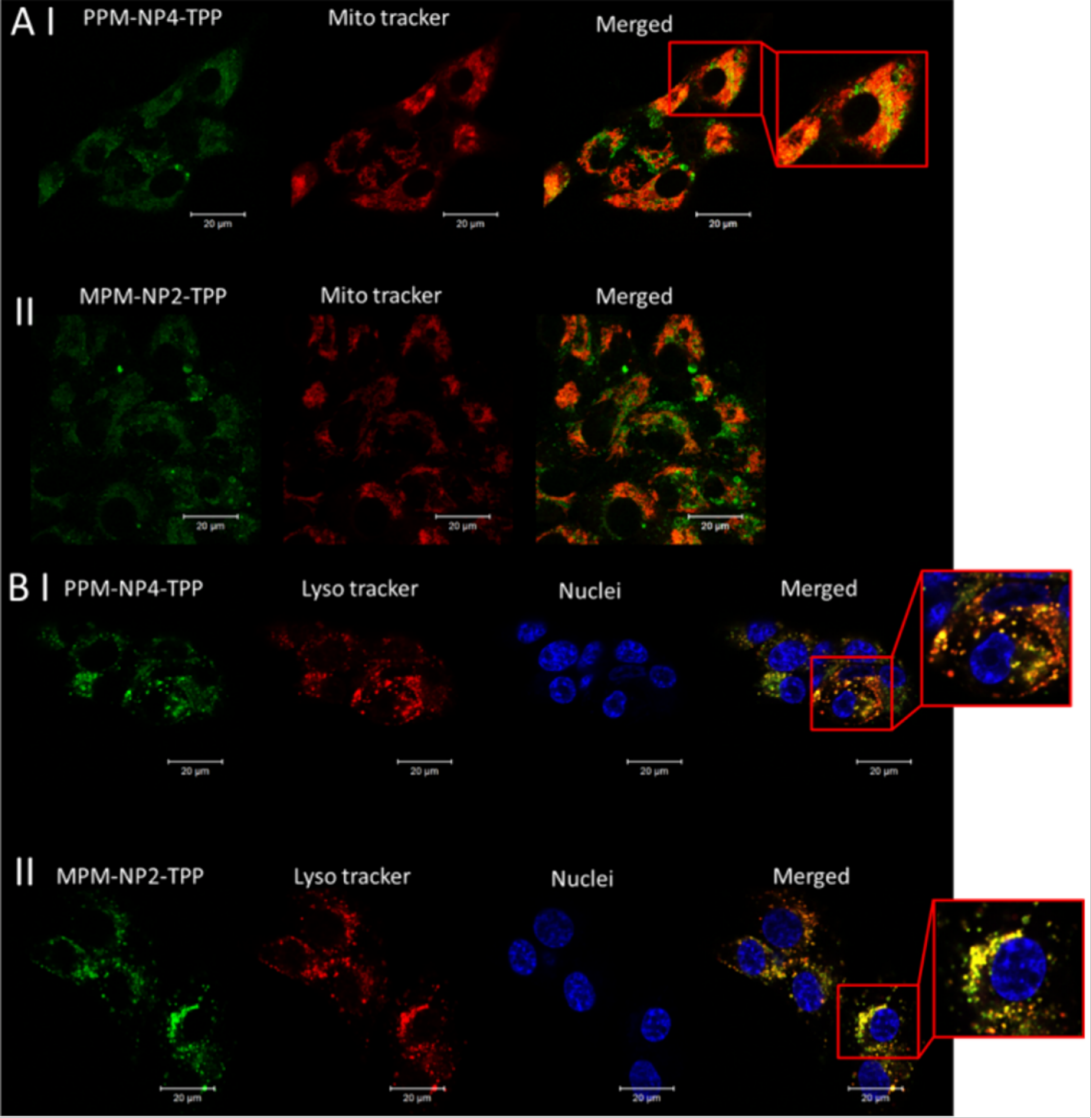
Cell localization of **PPM-NP4-TPP** (I) and **MPM-NP2-TPP** (II) into (A) mitochondria and (B) lysosomes und nuclei of SW982 cells. The particles carry fluorescein (green), the mitochondria and lysosomes were stain with Mito and Lyso Tracker, respectively (red) and the nuclei was stained with Hoechst 33342 (blue). Merged images show co-localisation (yellow fluorescence).

The co-localization of the TPP-variants into the mitochondria, lysosomes and nuclei were analysed using laser scanning confocal microscopy. Surprisingly, there was no improvement in mitochondria localization for the employed samples, despite carrying a mitochondria target on its surface according to the the Pearson correlation coefficient shown in Table 3. Moreover, **PPM-NP4-TPP** represents notable lower co-localization into the mitochondria than **PPM-NP4** (Figure 6 and Supporting Information File 1, Figure S2), which is supported by the calculation of the Pearson’s correlation coefficient (Table 3). The Pearson’s correlation coefficient – which is a statistical formula that calculates the correlation between two variables – decreases in number for the **PPM-NP4-TPP** micelles, indicating that less nanoparticles were accumulated within the mitochondria. It also shows a slightly lower number for the localization into the lysosomes, stating that overall lower cell internalization was achieved by the TPP-conjugated PEG micelle. Also the zwitterionic TPP-micelle led to no enhanced accumulation into the mitochondria (Figure 6 and Table 3) after 3 hours of incubation. However, an increase in lysosomal localization was observed and calculated. No co-localization was detected within the nuclei for both particle systems.

**Table 3:** Summary mitochondrial and lysosomal co-localization of **PPM-NP4**, **PPM-NP4-TPP**, **MPM-NP2** and **MPM-NP2-TPP** in SW982.

Organelles	Particles	Overlap coefficient^a^	Correlation R^b^	Correlation R × R^c^

mitochondria	**PPM-NP4**	0.62	0.10	0.01
	**PPM-NP4-TPP**	0.56	0.04	0
	**MPM-NP2**	0.43	−0.04	0
	**MPM-NP2-TPP**	0.38	−0.13	0.02

lysosomes	**PPM-NP4**	0.79	0.56	0.31
	**PPM-NP4-TPP**	0.77	0.48	0.23
	**MPM-NP2**	0.80	0.52	0.27
	**MPM-NP2-TPP**	0.84	0.63	0.4

^a^Manders overlap coefficient, ^b^Pearson’s correlation coefficient, ^c^Coefficient of determination.

**Figure 7:**
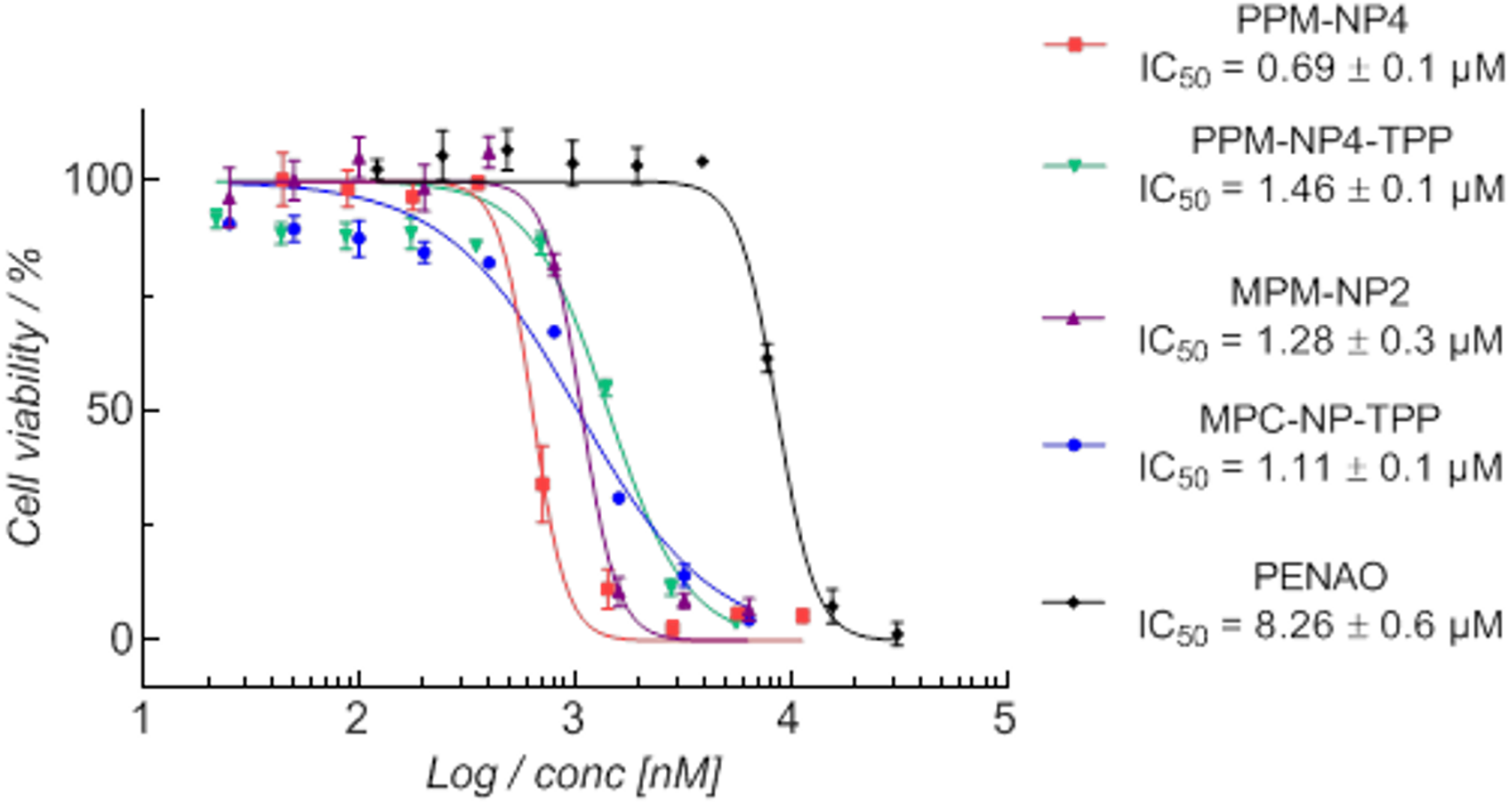
Cytotoxicity study of **PPM-NP4-TPP** and **MPM-NP2-TPP** on SW982 cells in relation to the concentration of PENAO present. The results of the nanoparticles without TPP have been reported earlier [42]. This figure was adapted with permission from [42], Noy et al., Direct Comparison of Poly(ethylene glycol) and Phosphorylcholine Drug-Loaded Nanoparticles In Vitro and In Vivo, *Biomacromolecules* **2020,** *21,* 2320–2333. Copyright 2020 American Chemical Society. This content is not subject to CC BY 4.0.

It seems therefore that TTP conjugation reduces the uptake by PEG-coated nanoparticles, but slightly, although not significantly increases the uptake in pMPC particles. We hypothesize that in both particles the nanoparticles remain in the lysosomes, but do not seem to escape the lysosomes to reach the mitochondria. However, the particles were still found to be active and inhibit the cell proliferation of human synovial sarcoma SW982 cells (Figure 7). All four PISA particles displayed an enhanced cytotoxicity compared to free PENAO using SW982. While PENAO’s cytotoxicity arises mainly from crosslinking two cysteine loops in the mitochondrial ANT protein, the here employed systems seem to not only rely on that reaction to introduce cell apoptosis, as all particle systems are cytotoxic regardless of reaching the mitochondria or not. The mitochondria contain indeed several vicinal protein thiols that readily react with trivalent arsenicals, however, it has been shown that various other proteins, enzymes and receptors bind to As(III) molecules [8–9]. It is on the other hand interesting that the zwitterionic micelles represent overall better anticancer efficiency when TPP is attached, while the PEG micelle performance is better without added TPP. The **PPM-NP4-TPP** micelle represents more than a 2-fold decrease in cytotoxicity, thus being less toxic compared to **PPM-NP4** micelle, while **MPM-NP2-TPP** displays slightly lower IC_50_ values than that of **MPM-NP2** (Figure 7). This is in agreement with the uptake results as **PPM-NP4-TPP** displays lower uptake while **MPM-NP2-TPP** displays higher uptake compared to the TPP-free PISA nanoparticle. It needs to be considered that these results here are unique to this cell line and different results can be obtained when using other cell lines. Such a study should include healthy cell lines such as cell lines of the immune system to ensure that the mitochondria binding ligand does not induce any damaging effects to these cell lines.

Many studies have reported that attaching targeting ligands on nanoparticle or liposome surfaces play a key role in overcoming biological barriers and reducing targeting effects. Active targeting or retention can increase cellular internalization as well as accumulation at the diseased tissues by targeting specific over-expressed receptors in cancer cells. However, to this date it is still debateable if active retention truly causes this "homing" effect. It has been shown that due to increased protein corona formation on a target agent carrying nanoparticle surface, the interaction between ligand and targeting agent is inhibited and resulted consequently in significant lower nanoparticle targeting efficiency [48–49].

This study demonstrates that every small change to nanoparticle design can result in unpredictable outcomes, illustrating the complexity in understanding and designing efficient drug-delivery systems in biological environments. It needs to be considered that TPP, despite its positive charge, still carries large phenyl groups that render the surface hydrophobic. While this can affect protein adsorption, it is more important to understand how TPP is presented on the surface of these PISA particles. In order for TPP to fulfil its function, the surrounding of the ligand needs to be considered. Cartoons are widely used in the drug delivery literature, often showing the targeting ligand readily available above a micelle shell of ordered polymer brushes. This assumes that the targeting ligand is well protruding out of the surface, which is usually not the case when the ligand was attached to endfunctionality of a micelle and is not attached to a longer chain. Chan and co-workers have shown that the binding of biorecognition molecules on a PEG nanoparticle to its target only works when the PEG chain length is less than the polymer chain to which the ligand is attached. Otherwise, the long PEG chain will intercept ligand binding [50]. It is also reasonable to think that TPP is barely visible on the surface at all. The hydrophobic phenyl group might redirect towards the hydrophobic core, disappearing into the polymer shell. Moreover, the targeting ligand might introduce changes to the shell structure that is not evident with the characterization carried out here. It has been shown that the presence of hydrophobic groups in the surface can reduce the hydration and limit cellular uptake [51–52]. It is therefore evident that more studies such as in-depth scattering studies are needed to fully elucidate the structure of the micelle [53]. Although TPP is present, it may not be fully available on the surface to display a significant mitochondria targeting effect.

## Conclusion

A triphenylphosphonium (TPP) mitochondria agent was attached to PISA nanoparticles with the aim to improve overall mitochondrial accumulation and therefore anticancer efficiency. However, having TPP on the nanoparticle surfaces only enhanced tumor penetration and cytotoxicity for the zwitterionic micelles, while no positive effect was seen for the PEGylated micelles. More importantly, no increased mitochondria targeting ability was observed for both micelles. While the attachment of TPP clearly influenced the biological behaviour, this behaviour may simply stem from the fact that TPP interacts with the shell or, thanks to the hydrophobic phenyl groups, even with the core of the micelle. More in-depth studies are necessary to answer the question if TPP is readily available on the surface of the micelle and if the presence of the polymer around TPP may interfere with binding to the mitochondria.

## Experimental

The synthesis of polymers and PISA particles and the biological experiments are described elsewhere [28,42], but the procedure has been added to Supporting Information File 1 for convenience.

### Attachment of TPP-COOH to **PPM-NP4** and **MPM-NP2**

In a typical experiment, (4-carboxybutyl)triphenylphosphonium bromide (TPP-COOH) (0.10 mg, 0.00022 mmol, 5 equiv to RAFT end group) was added to a 5 mg mL^−1^ particle solution before *N*-(3-dimethylaminopropyl)-*N*′-ethylcarbodiimide hydrochloride (EDC·HCl) (0.034 mg, 0.00022 mmol, 5 equiv to RAFT end group) and *N*-hydroxysuccinimide (NHS) (0.025 mg, 0.00022 mmol, 5 equiv to RAFT end group) were added (stock solutions were made in Milli-Q water and 25 µL of stock solution were added accordingly). The pH was corrected to 5.2 using 0.1 M NaOH or 0.1 M HCl solution. The solution was stirred for 45 min and the pH was adjusted to 8.3 and the solution was stirred for 3 days at room temperature. The particles were purified by dialysis in Milli-Q water with frequent solvent change (regenerated cellulose membranes, MW cut-off 6000–8000 g mol^−1^) and analysed via DLS experiments and TEM microscopy.

### Control experiment: attachment of TPP-COOH to **PP3** copolymer

For the control experiment, **PP3** (19.30 mg, 0.85 mmol of RAFT end group, 1 equiv) was dissolved in 1.5 mL of Milli-Q water and TPP-COOH (1.88 mg, 4.25 mmol, 5 equiv to RAFT end group), EDC·HCl (0.82 mg, 4.25 mmol, 5 equiv to RAFT end group) and NHS (0.49 mg, 4.25 mmol, 5 equiv to RAFT end group) were added to the polymer solution (the educts were added as stock solutions in Milli-Q water (100 µL), respectively) and the pH was adjusted to 5.2 using 0.1 M NaOH or 0.1 M HCl solution. After 45 min, the pH was corrected to 8.2 and the reaction was left to stir at room temperature for 3 days. The polymer was purified by dialysis in Milli-Q water with frequent solvent change (regenerated cellulose membranes, MW cut-off 6000–8000 g mol^−1^) and the product was then lyophilized and analysed using NMR spectroscopy.

^1^H NMR (300 MHz, D_2_O) δ_H_ (ppm) 8.0 (1H, aromatic RAFT), 7.8–7.9 (7 × 4H, aromatic PENAO and 15H TPP), 7.7 (2H, aromatic RAFT), 7.55 (2H, aromatic RAFT), 4.1–4.2 (36 × 2H + 2H, C*H_2_*-C=O of p(PEGMA) and RAFT agent), 3.80 (36 × 2H, C*H_2_*-CH_2_-O), 3.72 (36 × 2H × (~5)H + 7H, C*H*-N of PENAO and C*H*_2_-O of p(PEGMA)), 3.64 (2 × 7H, S-C**H**_2_-C=O of PENAO), 3.46 (36 × 3H, OCH_3_), 1.7–2.1 (140H, CH_2_ backbone), 1.5 (7 × 6H, CH_3_ PENAO), 0.7–1.2 (210H, CH_3_ backbone).

## Supporting Information

File 1 Analytical techniques, in vitro experiments, polymer synthesis, analysis of critical micelle concentration, and fluorescence microscopy of non-TPP micelles.
