# Supplementary material for: Post-functionalization of drug-loaded nanoparticles prepared by polymerization-induced self-assembly (PISA) with mitochondria targeting ligands
Source: Beilstein J Org Chem. 2021 Sep 3;17:2302–14. doi: 10.3762/bjoc.17.148 (PMC8450966; doi:10.3762/bjoc.17.148)
Supplement: File 1 — Analytical techniques, in vitro experiments, polymer synthesis, analysis of critical micelle concentration, and fluorescence microscopy of non-TPP micelles. [file Beilstein_J_Org_Chem-17-2302-s001.pdf]

BEILSTEIN JOURNAL OF ORGANIC CHEMISTRY

Supporting Information

for

Post-functionalization of drug-loaded nanoparticles prepared by polymerization-induced self-assembly (PISA) with mitochondria targeting ligands

Janina-Miriam Noy, Fan Chen and Martina Stenzel

Beilstein J. Org. Chem. **2021,** *17,* 2302–2314. doi:10.3762/bjoc.17.148

Analytical techniques, in vitro experiments, polymer synthesis, analysis of critical micelle concentration, and fluorescence microscopy of non-TPP micelles

License and Terms: This is a supporting information file under the terms of the Creative Commons Attribution License (https://creativecommons.org/licenses/by/4.0). Please note that the reuse, redistribution and reproduction in particular requires that the author(s) and source are credited and that individual graphics may be subject to special legal provisions.
The license is subject to the *Beilstein Journal of Organic Chemistry* terms and conditions: (https://www.beilstein-journals.org/bjoc/terms)

Materials

All chemicals were purchased as reagent grade from Sigma-Aldrich and used as received unless stated otherwise. Milli-Q water was produced by a milli-Q water purification system and had a resistivity of 18.2 MΩ.cm. The monomer methyl methacrylate (MMA) was passed through basic aluminium oxide (Al_2O_3) to remove inhibitors before polymerization. Deuterated NMR solvents were purchased from Cambridge Isotope Laboratories. Sarcoma cell lines (SW982 and 143B) were from one of our collaborators Prof. Jia-Lin Yang's laboratory at Sarcoma and Nano-oncology Group (Lowy Cancer Research Centre).

Analysis techniques

Nuclear magnetic resonance (NMR).

As described in ref. [1], NMR spectroscopic measurements were performed on either a Bruker Avance III 300 MHz (^{1}H: 300.17 MHz, ^{13}C: 75.48 MHz) or a Bruker Avance III HD 400 MHz (^{1}H: 400.13 MHz, ^{13}C: 100.62 MHz) instrument. Measurements of polymers were done in D_2O or MeOH-d_4. The internal solvent signal $\delta(D_2O)$ = 4.79 ppm or δ(MeOH-d_4) = 3.31 ppm was used as reference. NMR spectra were processed using either the Bruker TOPSPIN 3.2 software or MestReNova NMR software.

Dynamic light scattering (DLS).

As described in ref. [1], DLS was performed on aqueous solutions (approximately 1 mg mL^{-1} polymer in milli-Q water) which were analysed by a Malvern Zetasizer Nano ZS instrument equipped with a 4 mV He-Ne laser operating at λ = 632 nm and non-invasive backscatter detection at 173°. Measurements were carried out in a disposable cuvette at 25 °C, provided 15 scans equilibration period prior to each set of measurements. For a given sample, a total of three measurements were conducted with the number of runs, attenuator, and path length being automatically adjusted by the instrument, depending on the sample quality.

Critical micelle concentration (cmc)

The cmc was obtained by dynamic light scattering measurements, quantifying the scattering intensity for different micelle concentrations. The aqueous micelle solutions (approx. 1 mg mL^{-1}) were serially diluted with milli-Q water to 0–100 µg mL^{-1} working solutions. For each sample, a total of two measurements with a 10 scan equilibration period prior to each set of measurements were conducted. The number of runs was set to 11 (run duration 10 sec.), attenuator and path length was fixed to 7 and 4.65.

Laser scanning confocal microscopy (LSCM)

As described in ref. [1], LSCM (Zeiss LSM 780) was used to observe the cellular localization and spheroid penetration of the PENAO micelles. The system equipped with a Diode 405-30 laser, an argon laser and a DPSS 561-10 laser (Excitation and absorbance wavelengths: 405 nm, 488 nm and 633 nm, respectively) connected to a Zeiss Axio Observer Z1 inverted microscope (oil immersion x 100/1.4 NA objective). The ZEN2011 imaging software (Zeiss) was used for image acquisition and processing.

Statistical analysis

As described in ref. [1], all data for in vitro studies were reported was mean ± standard deviation (SD). A one-way analysis of variance was performed for the statistical analysis followed by a Turkey's post hoc test for pairwise comparison. A *P* value <0.05 was considered statistically significant. All statistical analysis was done with GraphPad Prism 7.

IC_{50} Determination via WST-1 assay

As described in ref. [1], cytotoxicity test of polymeric micelles **PPM-NP4**, **MPM-NP2** and PENAO were determined by a WST-1 based assay. Synovial sarcoma (SW982) cell lines were seeded in a 96-well plate (8000 cells/well) and incubated at 37 °C in a 5% CO_2 atmosphere for 24 h. For the cytotoxicity assay, the medium in the cell culture plate was discarded and 100 µL of fresh 2x concentrated RPMI 1640 medium was added to each well of the 96-well culture plate. The specimens were sterilized by UV irradiation for 20 min before serially diluting (sequential halved dilution) with sterile water. They were then added into the plate at 100 µL per well, respectively. As a control, sterile water (100 µL) was added to the non-treated cells. The cells were incubated with the particles and PENAO for 72 h. The old medium was discarded, and cells were washed with PBS. 100 µL of fresh warm medium along with 5 µL WST-1 was added to each well, and the plates were incubated for further 3 h at 37 °C before reading the absorbance on a Bio-Rad BenchMark microplate reader at 440 nm with a reference wavelength of 650 nm. Dose-response curves were plotted accordingly where the values were

expressed as percentage of control (non-treated cells were used as controls). The optical density was used to calculate cell viability. The experiments were carried out in triplicate.

3D Sarcoma MCTS formation

As described in ref. [1], the sarcoma MCTSs were prepared by a liquid overlay method [2]. The 143B and SW982 cells were suspended in RPMI-1640 media at a density of 12.5 x 10^3 cells/mL and 15 x 10^3 cells/well respectively. The cells were seeded (200 µL) into each well of an ultralow attachment 96-well plate (Coming) and the plate was centrifuged for 5 min at 1900 rpm and incubated at 37 °C for 5 (143B) and 6 days (SW982). The spheroid formation was recorded by an inverted microscope with CCD camera (Leica).

Micellar treatment of sarcoma MCTS

As described in ref. [1], after the sarcoma MCTSs were formed, 150 µL of culture media was removed and replaced by 200 µL of fresh media supplemented with PENAO, **PPM-NP4**, **MPM-NP2**, **PPM-NP4-TPP** or **MPM-NP2-TPP** micelles (approx. total micelle concentration = 2 and 0.5 mg mL^{-1}) and incubated. The spheroid morphology was recorded with the microscope at day 3 and 6, and the cell viability of the treated MCTSs was determined by an acid phosphatase (APH) assay as described elsewhere after 6 days [3]. Briefly, the plates were centrifuged for 5 min at room temperature at 500 g to spin down spheroids, clusters, and single cells. 150 µL of the supernatant was then removed and the spheroids were washed carefully with 150 µL PBS. The centrifugation was repeated, and the spheroids were washed with PBS again. 150 µL of PBS was taken out and 100 µL of APH assay buffer solution was added to the remaining 50 µL in each well and incubated for 90 min at 37 °C and 5% CO_2. Each well was then supplemented with 10 µL of a 1 M NaOH solution, and the supernatant was transferred to standard flat-bottomed 96-well microplates, and the absorption was red at 405 nm on a microplate reader (Ascent) within 10 min. The APH assay buffer was composed of 0.1 M sodium acetate, 0.1% (v/v) Triton X-100 (pH 4.8), and 2 mg mL^{-1} *p*-nitrophenyl phosphate, which was added just before use.

Penetration studies into sarcoma MCTS

As described in ref. [1], the formed SW982 and 143B MCTSs were washed with 150 µL PBS as described above before the particles (100 µL mL^{-1} in 200 µL media) were loaded into each well and incubated for 3 hours at 37 °C and 5% CO_2. The spheroids were washed with Hanks' Balanced Salt Solution twice and imaged using a Zeiss LSM780 laser scanning confocal microscope. The excitation wavelengths were set as 488 nm and the images were captured and processed using ZEN software.

Particle localization via confocal microscopy

As described in ref. [1], SW982 cells were seeded into a fluoro dish at a density of 5 x 10^4 cells in 2 mL RPMI medium per dish and incubated at 37 °C at a 5% CO_2 atmosphere for 24 h. The medium was discarded, and the cells were washed with PBS (1 mL x 3) and treated with 100 µg mL^{-1} micelle solutions in 1 mL RPMI medium for 2 h. For mitochondrial localisation: The medium was discarded, and the cells were washed with Hanks' Balanced Salt Solution (HBSS, 1 mL x 3) before 1 mL Mito Tracker® Deep Red FM dye (500 nM in HBSS) was added. The cells were incubated for another 30 min with the dye and washed with HBSS (1 mL x 2). For lysosomal and nuclei localisation: The cells were washed with HBSS (1 mL x 3) and incubated with Hoechst 33342 (2 µg mL^{-1} in HBSS) for 5 min, washed with HBSS (1 mL x 3), and then incubated with LysoTracker™ Deep Red (100 nM in HBSS) for 1 min and washed with HBSS again (1 mL x 3) before images were taken using a Zeiss LSM780 confocal microscope. The excitation wavelengths were set as 488, 633 and 405 nm for micelles, MitoTracker/LysoTracker and Hoechst 33342 respectively. A 100 × oil objective lens (N.A = 1.4) was used and the images were captured and processed using ZEN software.

Synthesis of RAFT agent 2-hydroxyethyl 4-cyano-4-((phenyl-carbonothioyl)thio)pentanoate CPADB-OH

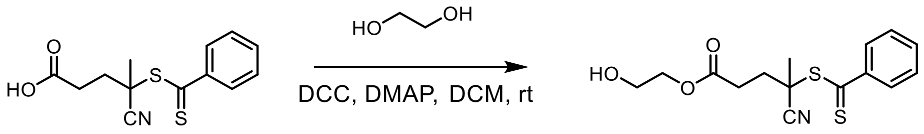

CPADB **CPADB-OH**

CPADB (1.00 g, 3.58 mmol, 1 equiv) was weight into a 50 mL three-necked flask and dissolved in 16 mL anhydrous dichloromethane under nitrogen atmosphere. DMAP (0.09 g, 0.72 mmol, 0.2 equiv), DCC (0.89 g, 4.30 mmol, 1.2 equiv) and then ethylene glycol (0.11 g, 0.10 mL, 1.79 mmol, 0.5 equiv) were added and the solution was stirred at room temperature. The reaction was controlled by TLC and showed complete reaction after 4 hours. The cloudy mixture was filtered and the solvent was removed under reduced pressure. The crude product was then purified by silica gel column chromatography using a 1:4 solvent mixture of ethylacetate/hexane. The product was obtained as pink sticky liquid (yield = 57%).

Synthesis of p(PEGMA-*co*-PENAO) copolymers (PP3).

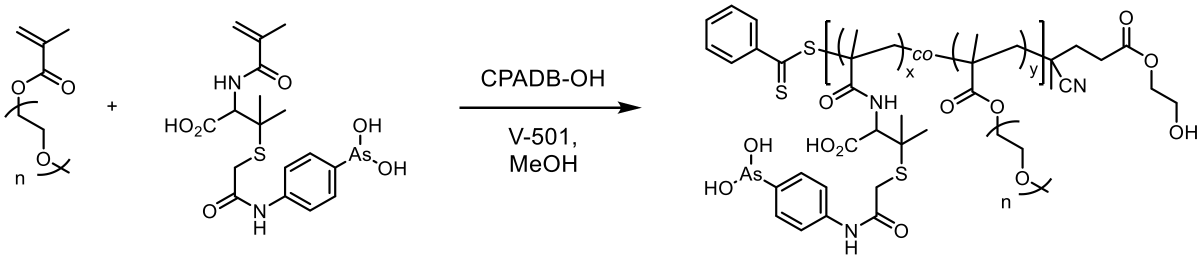

As described in ref. [1], PEGMA and PENAO MA were copolymerized using CPADB-OH as the chain transfer agent (CTA), V-501 as the initiator and fluorescein *O*-methacrylate as the fluorescent stain. The ratio of CTA, V-501 and fluorescein *O*-methacrylate were kept constant [CTA]:[V-501]:[fluorescein] = [1]:[0.2]:[0.3], while the ratios of PEGMA and PENAO MA was set to [PEGMA]:[PENAO MA] = [80]:[20] **PP3**. In a typical experiment PEGMA, PENAO MA, CTA, V-501 and fluorescein *O*-methacrylate were dissolved in methanol (c = 1.26–1.54 mol L^{-1}) in a glass flask. The flask was protected from light and equipped with a rubber septum. The clear solution was purged with nitrogen for 20–25 min, and then placed into a preheated oil bath at 70 °C for 5 h. The polymerization was stopped by quenching to room temperature and the monomer conversion and theoretical molecular weights, ($M_n^{theo.}$) were determined by ^{1}H NMR spectroscopic measurements of a reaction sample (50–100 µL) diluted with MeOD (450 µL) by comparison of polymeric signals with the signals of the residual monomer. The polymers were purified by dialysis in milli-Q water (regenerated cellulose membranes, MW cut-off 3500 g mol^{-1}).

^{1}H NMR (300 MHz, D_2O) δ_H (ppm) = 8.0 (1H, aromatic RAFT), 7.8-7.9 (7×4H, aromatic PENAO), 7.7 (2H, aromatic RAFT), 7.55 (2H, aromatic RAFT), 4.1-4.2 (36×2H+2H, C**H₂**-C=O of p(PEGMA) and RAFT agent), 3.80 (36×2H, C**H₂**-CH_2-O), 3.72 (36×2H×(~5)H+7H, C**H**-N of PENAO and C**H**$_2$-O of p(PEGMA)), 3.64 (2×7H, S-C**H**$_2$-C=O of PENAO), 3.46 (36×3H, OCH_3), 1.7-2.1 (140H, CH_2 backbone), 1.5 (7×6H, CH_3 PENAO), 0.7-1.2 (210H, CH_3 backbone)

Synthesis of p(MPC-*co*-PENAO) Copolymers (MP2).

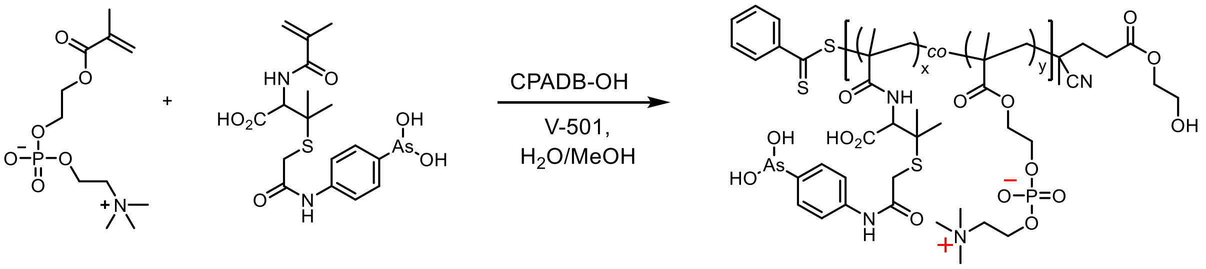

As described in ref. [1], the PENAO containing copolymer **MP2** was prepared by RAFT polymerization according to the following general procedure. MPC and PENAO MA (0.32 mmol in total), CTA (1 equiv), V-501 (0.2 equiv) and fluorescein *O*-methacrylate (0.3 equiv.) were dissolved in a 1:1 methanol / milli-Q water mixture (1.6 mL, approx. total concentration of 0.23 mol L^{-1}) in a glass flask which was equipped with a magnetic stir bar and sealed with a rubber septum. The solution was purged with nitrogen for 15–20 min and placed into a preheated oil bath at 70 °C for 3.5 h. The polymerization was stopped by quenching to room temperature. Monomer conversion and theoretical molecular weights, $M_n^{theo.}$, were determined by ^{1}H NMR spectroscopic measurement of a reaction sample (50 µL) diluted with D_2O (450 µL) by comparison of polymeric signals with the vinyl signals of residual monomers. The polymer was purified by dialysis in milli-Q water (regenerated cellulose membranes, MW cut-off 3500 g mol^{-1}).

^{1}H NMR (300 MHz, D_2O) δ_H (ppm) = 8.0 (1H, aromatic RAFT), 7.8-7.9 (4×4H, aromatic PENAO), 7.7 (2H, aromatic RAFT), 7.55 (2H, aromatic RAFT), 4.33 (17×2H, O-C**H₂**-CH₂-N⁺), 4.25 (17×2H, C**H₂**-O-C=O), 4.12 (4×2H, C**H₂**-O-P), 3.6-3.65 (4×2H, C**H**-N of PENAO, 17×2H, O-CH₂-C**H₂**-N⁺ and 4×2H S-C**H₂**-C=O of PENAO), 3.26 (4×9H, NCH_3), 1.7-2.1 (42H, CH_2 backbone), 1.5 (4×6H, CH_3 PENAO), 0.7-1.2 (63H, CH_3 backbone)

PPM-NP Particle formation via polymerization-induced self-assembly (PISA).

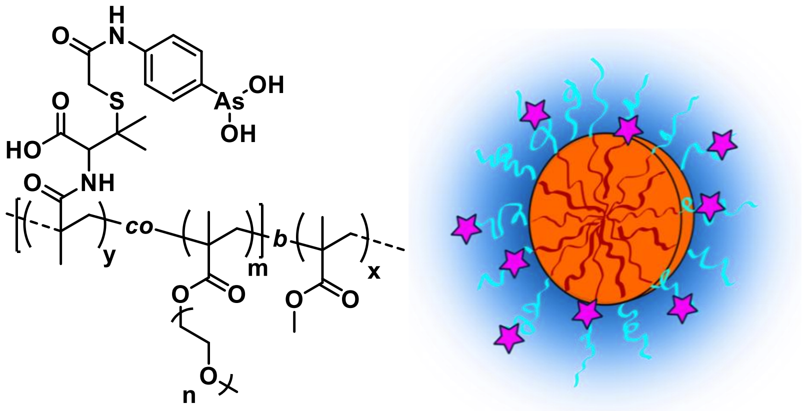

The previous described copolymers **PP3** and **MP2** were used as macro RAFT agents for the particle formation by the PISA process with methyl methacrylate (MMA). As described in ref. [1], in a typical experiment the macro RAFT agent **PP3** or **MP2** (1 equiv), MMA (1000, 1500 or 5000 equiv), and initiator V-501 (0.5 equiv) were dissolved in methanol (approx. total concentration of 0.50 mol L^{-1}) in a glass flask equipped with a septum and magnetic stir bar. The absolute concentrations are listed in the Table below. Trioxane (0.05 equiv to

MMA) was added as internal standard to the solution and 50 µL of the reaction sample was withdrawn, diluted with MeOD (450 µL), and analyzed by ^{1}H NMR spectroscopy. The remaining reaction mixture was protected from light, purged with nitrogen for 20–25 min and placed into a preheated oil bath at 70 °C for 4.5–6 h. The polymerization was stopped by quenching to room temperature. Monomer conversion and theoretical molecular weights, $M_n^{theo.}$, were determine by a ^{1}H NMR spectroscopic measurement of reaction sample (50 µL) diluted with MeOD (450 µL) by integration of the internal standard trioxane to 1 and the monomer peaks before and after polymerization were compared. The particles were first purified by dialysis in methanol for 1 day with frequent solvent change and the solvent was then switched to pure Milli-Q water and dialysed for a further 2 days with frequent water change (regenerated cellulose membranes, MW cut-off 6000–8000 g mol^{-1}).

MPM-NP Particle formation of MPC micelles via polymerization-induced self-assembly (PISA)

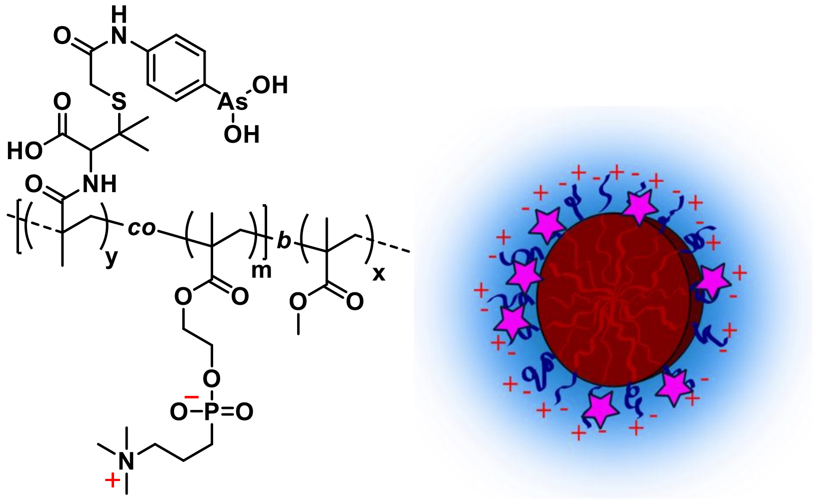

As described in ref. [1], **MP2** (5.00 mg, 0.00070 mmol, 1 equiv), MMA (69.75 mg, 0.70 mmol, 1000 equiv), V-501 (0.10 mg, 0.00035 mmol, 0.5 equiv) and NMR standard trioxane (3.14 mg, 0.035 mmol, 0.05 equiv to MMA) for **MPM-NP1** and **MP2** (5.00 mg, 0.00070 mmol, 1 equiv), MMA (104.63 mg, 1.05 mmol, 1500 equiv), V-501 (0.10 mg, 0.00035 mmol, 0.5 equiv) and NMR standard trioxane (4.71 mg, 0.052 mmol, 0.05 equiv to MMA) for **MPM-NP2** were dissolved in a Milli-Q : ethanol mixture (total 1.4 mL (**MPM-NP1**) and 2.1 mL (**MPM-NP2**), approx. total concentration of 0.50 mol L^{-1}, Milli-Q water : ethanol, 60:40 vol.%) and 50 µL of the reaction sample were withdrawn, diluted with D_2O (450 µL), and analysed by ^1H NMR spectroscopy. The remaining reaction mixture was protected from light, purged with nitrogen for 20–25 min and placed into a preheated oil bath at 70 °C for 6 h. After cooling, the conversion was obtained via ^1H NMR spectroscopy by diluting 50 µL of the milky reaction mixture with D_2O (450 µL) and comparing the monomer peaks via the integration of the internal standard trioxane before and after polymerization. The particles were purified by dialysis in milli-Q water with frequent solvent change (regenerated cellulose membranes, MW cut-off 6000–8000 g mol^{-1}) and analysed via dynamic light scattering and TEM microscopy.

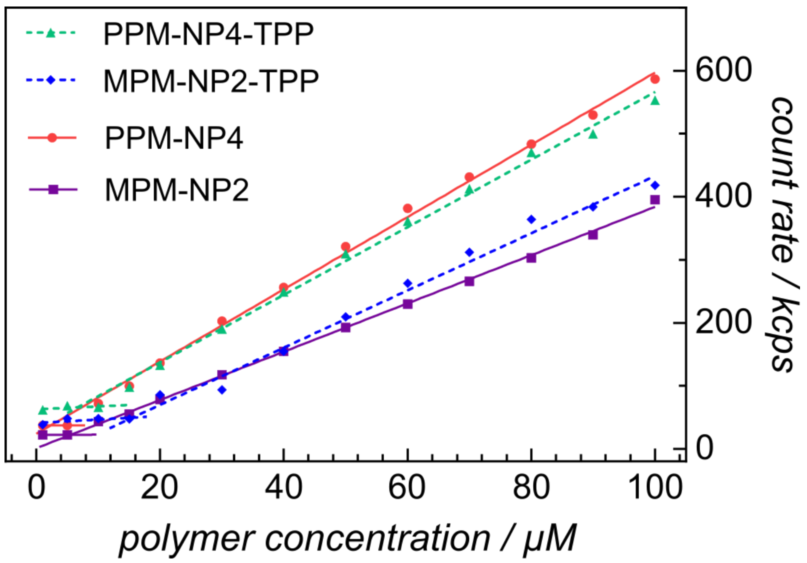

Figure S1. Scattering intensity *vs*. concentration plots for cmc determination of drug-directed PISA nanoparticles before (**PPM-NP4**, **MPM-NP2**) and after (**PPM-NP4-TPP**, **MPM-NP2-TPP**) TPP attachment. The results of the nanoparticles without TPP have been reported earlier[1] and they are reprinted (adapted) with permission Noy et al, Direct Comparison of Poly(ethylene glycol) and Phosphorylcholine Drug-Loaded Nanoparticles In Vitro and In Vivo, Biomacromolecules 2020, 21, 2320-2333. Copyright 2020 American Chemical Society

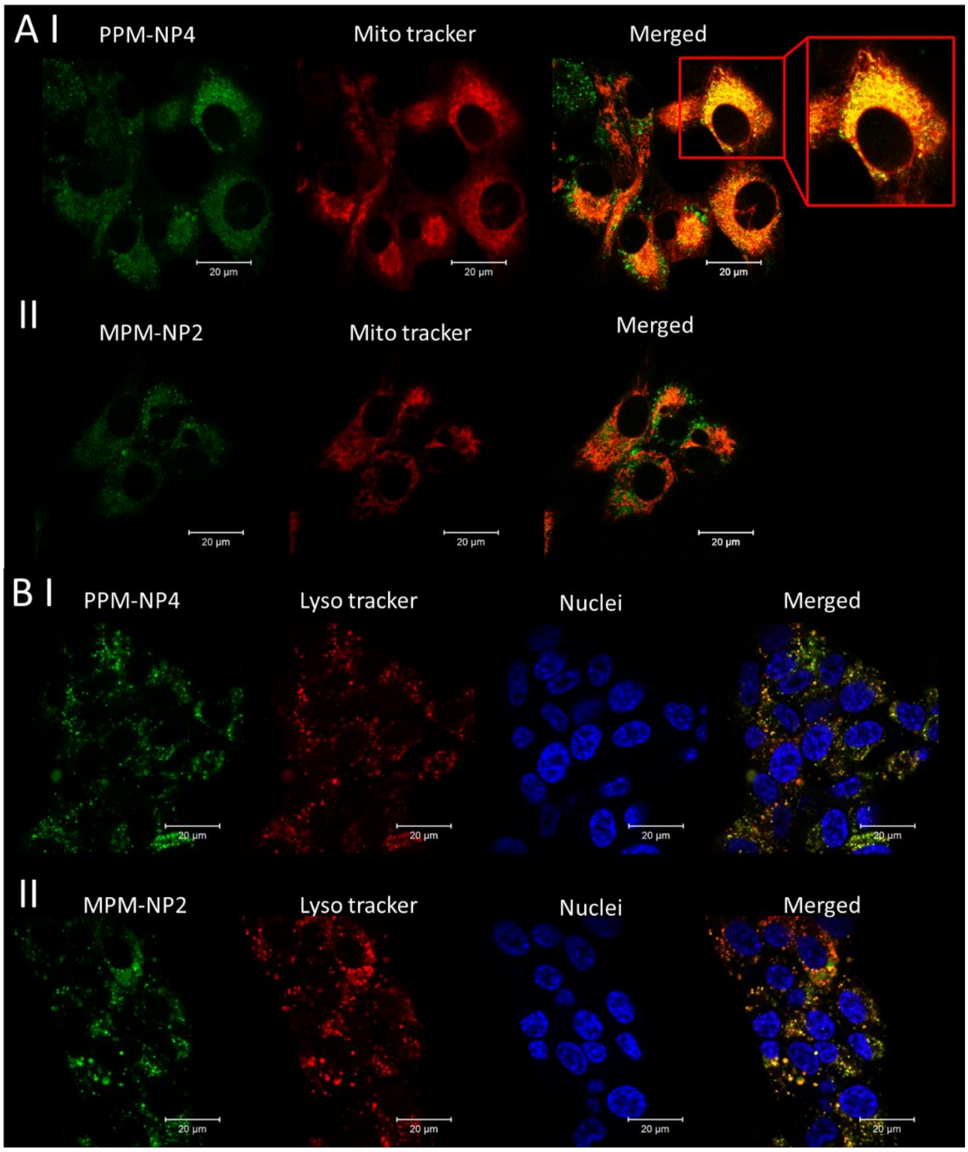

Figure S2. Cell localization of **PPM-NP4** (I) and **MPM-NP2** (II) into (A) mitochondria and (B) lysosomes und nuclei of SW982 cells. The particles carry fluorescein (green), the mitochondria and lysosomes were stain with Mito and Lyso Tracker, respectively (red) and the nuclei was stained with Hoechst 33342 (blue). Merged images show co-localisation (yellow fluorescence). This figure was reprinted with permission from [1], Noy et al, Direct Comparison of Poly(ethylene glycol) and Phosphorylcholine Drug-Loaded Nanoparticles In Vitro and In Vivo, Biomacromolecules 2020, 21, 2320-2333. Copyright 2020 American Chemical Society. This content is not subject to CC BY 4.0

References

1. Noy, J.-M.; Chen, F.; Akhter, D. T.; Houston, Z. H.; Fletcher, N. L.; Thurecht, K. J.; Stenzel, M. H., *Biomacromolecules* **2020,** *21*, 2320-2333.

2. Lin, R. Z.; Chang, H. Y., *Biotechnol. J.* **2008,** *3*, 1172-1184.

3. Friedrich, J.; Seidel, C.; Ebner, R.; Kunz-Schughart, L. A., *Nat. Protoc.* **2009,** *4*, 309-324.
